# Why Is It Difficult to Diagnose My Child’s Asthma? A Patient Physician Perspective of Asthma Management

**DOI:** 10.1007/s41030-019-0094-x

**Published:** 2019-07-03

**Authors:** Kerri Jones, Prasad Nagakumar, Satish Rao

**Affiliations:** 1 Bournemouth, UK; 2 Department of Paediatric Respiratory Medicine and Cystic Fibrosis, Birmingham Women’s and Children’s Hospital, Birmingham, UK

**Keywords:** Asthma, Asthma management, Patient

## Abstract

This article is co-authored by the mother of a patient living with asthma, and two consultants in respiratory medicine from Birmingham Women’s and Children’s Hospital. The commentary article describes the mother’s experience of the diagnosis and treatment process of her son’s asthma. The consultants then discuss paediatric asthma diagnosis and management in the context of the patient’s experiences.

## Patient’s Experience: Mother’s Perspective

Ethan is a stoic character, never one to complain of feeling unwell. So it came as a surprise when, having been fighting off a cold, he suddenly developed a persistent dry cough and was struggling to talk without gasping for air. He was almost 3 years old and like most young children, he constantly endured coughs and colds. However, these symptoms were new. The GP diagnosed a chest infection and prescribed antibiotics and a Ventolin inhaler. I assumed only people with asthma used inhalers. I didn’t question it though; I had no medical training. I was advised to “give him two puffs if he needs it”. She didn’t seem too concerned. I filled the prescription and the pharmacist showed me how to use the inhaler with a spacer. I was confident Ethan would be fine with a couple of puffs now and again. I had no idea how poorly he really was.

By some miracle Ethan improved over the following days. We didn’t go to hospital but we should have; I didn’t know his condition was life threatening. Had the GP showed more concern, we would have gone straight to hospital. The guilt I felt for not getting him the help he needed still troubles me. He was so sick and I had failed him.

Some months later it happened again, but this time he hadn’t been poorly beforehand. Ethan struggled to talk, had a persistent dry cough and was despondent and lethargic. The GP surgery was closed for bank holiday Monday. I phoned NHS Direct for an out of hours GP appointment but was told to take him straight to A&E or to phone for an ambulance. That first visit to hospital was terrifying. There were doctors and nurses and tests and medications and we didn’t know what was going on.

Twenty-four hours, two nebulisers, some prednisolone and a lot of Ventolin later, Ethan was much improved. A doctor explained Ethan probably had an asthma attack. He had a wheeze, his saturation dropped to 88, and he was sucking his tummy in under his ribs where he was working so hard to breathe. I relayed our previous experience with the GP. The doctor explained how difficult it is to diagnose asthma in young children, as symptoms can mimic other illnesses, such as a chest infection. I understood, but I was disappointed that a more thorough investigation wasn’t carried out at our first GP visit. She didn’t check if he was sucking in his tummy, she didn’t check his oxygen saturations; she just listened to his chest, which led to an insufficient and possibly incorrect conclusion.

Over the following months, we had multiple GP appointments—seeing a different one each time—and several hospital admissions. We learnt more about his condition with each episode. Other parents at the hospital shared similar experiences of their quest to obtain a proper diagnosis (be it asthma or otherwise) and appropriate treatment for their children.

Ethan was finally diagnosed with asthma 9 months after the first attack. He was prescribed a Clenil inhaler to be used twice daily and we were to continue with Ventolin as needed. We saw an asthma nurse who was fantastic. She showed us how to use Ethan’s inhalers properly and wrote him an asthma action plan. We learnt his asthma is exacerbated by viruses and is worse during the spring when the pollen count is high. We were told to start Ventolin as soon as Ethan shows signs of a virus, starting with two puffs, gradually increasing the dose as the virus progresses and then reducing it again as he gets better. He was also advised to take antihistamine in the spring months and we had an emergency supply of prednisolone to keep at home.

Ethan isn’t a complainer. It means we can’t rely on him to tell us when his asthma flares up. I worried about him managing his asthma when he started school. At 4 years of age, he wouldn’t have told his teacher if his asthma was bad. He couldn’t describe his symptoms. He would say his mouth was sore and his tummy ached. In reality, his throat was sore and his lungs ached from the effort he was making to breathe. I gave the school Ethan’s asthma action plan and an inhaler to be kept in the first aid room. Thankfully in those early years, he didn’t need either. He is eight now and keeps his inhaler with him. However, he doesn’t always use it when he should. He recently had his first serious asthma attack in over 18 months. It started at school but he didn’t tell anybody because he didn’t want to go home; he wanted to play football with his friends at break time.

I tell Ethan how important it is to get help when needed and that if he doesn’t, it could be fatal. But at 8 years old, he thinks he’s invincible. He’s unable to comprehend how serious his condition is if not treated properly. So now when Ethan is ill, we ask him to describe how he is feeling. We don’t say “are you ok?” because his answer will invariably be “yes”, regardless. I also have to monitor his SATs and peak flow and I check he isn’t sucking his tummy under his ribs; all classic “Ethan symptoms”. I know if his oxygen saturation are below 96, he needs Ventolin. If his oxygen saturation are below 92 and peak flow below 130, he needs to go to hospital, because he’s not going to get better at home.

In the last 5 years Ethan’s medication has been adjusted several times. Clenil was replaced with Seretide and he takes montelukast daily. He only needs Ventolin occasionally and Prednisolone rarely.

There’s no failsafe way to diagnose asthma and it is particularly difficult in children. Advice given by clinicians is often inconsistent and, sadly, much of the learning process is trial and error; it seems children often suffer several attacks before they get appropriate support.

## Physician’s Response

### Why Is It Difficult to Diagnose My Child’s Asthma?

We thank Ethan’s mother for sharing his asthma journey. Asthma is the most common long-term health condition in childhood. One in 11 children in the UK have the diagnosis of asthma [1]. Asthma is a heterogenous inflammatory condition of the lungs manifesting by wheeze, cough and breathlessness [2]. Proper use of regular inhaled steroids reduces the inflammation and thereby improves the asthma symptoms. The National Institute of Clinical Excellence (NICE) [3] guidelines recommend the use of the following parameters for the diagnosis of asthma: history of wheeze, peak flow variability of > 20%, spirometry showing evidence of airway obstruction, bronchodilator reversibility of > 12% and/or high exhaled nitric oxide (FeNO) > 35 parts/billion. The difficulty in performing these tests routinely, particularly in those < 6 years old, remains a challenge. Unfortunately, in children, the assessment of response to a trial of inhaled steroids and salbutamol remains a common method of diagnosing asthma.

### “Asthma” in Young Children

Ethan’s mother has clearly described her frustrations and difficulties in getting Ethan’s diagnosis of asthma when his symptoms started at 3 years of age. The health care professionals are faced with several factors whilst diagnosing asthma in young children.

Firstly, Speight et al. [4] described the reluctance of health care professionals to diagnose asthma in young children resulting in needless morbidity. Recently, concerns have been raised regarding “over diagnosis” of asthma in young children resulting in excessive prescription of inhalers [5]. Over prescription of inhalers may result in asthma not been taken seriously, and resources and expertise in asthma care being diluted. Of note, at present there are no interventions available to prevent a child developing asthma, nor there is evidence that early initiation of treatment will change the likelihood of viral wheeze changing into asthma [6].

Secondly, not all children who have pre-school wheeze develop asthma in the future. Whilst a third of preschool children develop wheeze with viral infections [7], the majority of these children do not have or develop “asthma” in the future. Other epidemiological studies have also shown that a large proportion of pre-school children with wheeze (episodic viral wheeze) resolve spontaneously by school age.

Thirdly, unlike conditions like diabetes, where a simple blood test can establish the diagnosis, asthma in children is predominantly diagnosed based on a constellation of symptoms. Although spirometry can be performed in 2- to 5-year-olds in a research setting [8], there are no specific tests which can be performed routinely in primary care to help diagnose asthma in young children. It is important to remember that not all wheeze is asthma and several other conditions can cause wheeze or noisy breathing (Table 1). These factors often can make the diagnosis of asthma in young children difficult.

**Table 1:** Not all wheeze is asthma, and several other conditions can cause wheeze or noisy breathing

Congenital structural airway disease	Complete cartilage rings, cysts, webs
Upper airway disease	Adenotonsillar hypertrophy, rhinosinusitis, subglottic stenosis, laryngomalacia
Bronchial/tracheal compression	Vascular rings and sling, enlarged heart or lymph nodes
Endobronchial disease	Foreign body, tumour
Oesophageal/swallowing problems	Reflux, incoordinate swallow, laryngeal cleft or tracheo-oesophageal fistula
Suppurative lung disease	Cystic fibrosis, primary ciliary dyskinesia, persistent bacterial bronchitis, immunodeficiency

The diagnosis of asthma is further complicated as the word “wheeze” is often used to describe other types of noisy breathing, too. Parents and carers often referred to the “rattly” noisy breathing caused by upper airway secretions as “wheeze” [9]. As it is not uncommon for the child to be asymptomatic when seen in the clinic, parental report of “wheeze” could result in both over-diagnosis and under-diagnosis of asthma. We recommend that the clinicians take time to describe that the term “wheeze” is referred to a whistling noise heard when the child is breathing out (expiration) to help parents identify the symptom accurately. It may also be helpful to look through the previous records for any evidence of “physician-diagnosed wheeze” which could make the diagnosis of wheeze more secure.

A child with wheeze heard on expiration in both the lung fields, along with presence of atopic conditions like eczema, allergies, wheeze on exercise and a family history of asthma, increases the possibility of asthma diagnosis. The clinical asthma prediction score (CAPS), a simple score ranging from 0 to 11 aimed at primary care physicians, can be used to inform the families about the likelihood of preschool wheeze developing into future asthma [10]. A score of ≥ 7 has high predictive value for future asthma, while a score ≤ 3 makes the risk of future asthma less likely. Often, however, the clinical improvement of the child with a trial of treatment with salbutamol and inhaled steroid seems to be the reliable way of diagnosing asthma in pre-school children.

Despite the difficulties in making a diagnosis of asthma at this age, prompt diagnosis allows institution of an acute asthma plan to help families manage acute asthma attack appropriately. In Ethan’s case, this could have helped in identifying the severity of the acute attack and perhaps referring to the hospital for further management early in the course of illness. It could have also allayed the anxieties of the family, as they could have been better prepared to manage an acute asthma attack. Use of a structured acute asthma plan empowers families in dealing with such emergencies.

### Asthma in School Age Children

In school age children, the diagnosis of asthma is much easier. The diagnosis is still made on a good clinical history: a child who is otherwise well, wheezes and coughs or becomes breathless with colds and/or exercise, and shows response to salbutamol inhaler. The British Thoracic Society (BTS) guidelines [11] recommend classifying children into those with high, intermediate and low probability of having asthma based on symptoms. Objective tests like peak flow monitoring (PFM) have been traditionally used in primary care for diagnosis of asthma. PFM is done twice daily for 2–4 weeks. A variability of peak flow of > 20% during the monitoring period is diagnostic of asthma. The BTS and the NICE guidelines recommend spirometry tests for diagnosis of asthma. A forced expiratory volume in 1 s (FEV1) and forced vital capacity (FVC) ratio of < 70% and/or bronchodilator reversibility (BDR) of > 12% are the diagnostic features of asthma. In BDR testing, FEV1 is measured and the subject is administered six puffs of salbutamol inhaler. The spirometry is repeated 10–15 min later and an improvement of FEV1 by > 12% is considered as one of the objective measurements for diagnosis of asthma. Although the test can be performed reliably in school aged children, health care professionals trained and experienced in performing lung function tests in children should be available to perform and interpret the tests. In primary care, and even in secondary care, it is still a challenge to get spirometry done in a school aged child in a timely manner. Recently, NICE also recommend measurement of exhaled nitric oxide (FeNO) to aid diagnosis of asthma in children older than 5 years. Levels > 35 parts/billion (ppb) suggest eosinophilic airway inflammation. Unlike adults, FeNO can be high in children due to non-asthmatic reasons, including technique of measurement, allergies, nasal symptoms and diet. FeNO on its own should not be used for either diagnosis or monitoring of asthma in children. As in pre-schoolers, a 6–8 weeks trial of treatment with asthma inhalers and response assessment is commonly used to diagnose asthma.

We would like to emphasise that as asthma is an inflammatory condition, any treatment should include inhaled steroids and not just a bronchodilator. Medications should be reviewed and stopped if there is no clinical response.

Once Ethan was diagnosed, he was managed appropriately with support from both primary and secondary care. The aim of effective asthma management is to achieve asthma control resulting in minimal symptoms during day and night, minimal need for the reliever inhaler, no asthma attacks and no limitation of physical activities. Good asthma control can be achieved by regular asthma reviews at the GP surgery, with emphasis on educating and re-educating children and carers about the importance of correct use of inhalers and adherence to prescribed medications.

Metered dose inhalers are preferred in children, and the inhalers should always be administered via a spacer device. Age appropriate spacer devices should be provided. Children older than 4 years should be prescribed a spacer device with a mouthpiece, not a mask. The Asthma UK have produced excellent videos which the families can access. The videos can also be played on the TV screens at the surgery waiting areas (https://www.asthma.org.uk/advice/inhaler-videos/). The importance of repeated education of the child, young person and families cannot be over-emphasised. In one of the studies, whilst a majority of the children using a metered dose inhaler with a spacer were able to demonstrate the right technique of use after one education session, it took at least three sessions with the dry powder device to achieve a similar level of competence [12].

A written asthma plan should be provided which includes advice to the families about when to seek medical help. Although acute asthma attacks can be life-threatening, seeking medical help at the right time is enough to keep the majority of the patients safe. Therefore, it is important to emphasise the potential seriousness of an acute asthma attack, together with effective self-management and recognition of the threshold for promptly seeking medical help.

In a child with poor asthma control, consider the following before increasing the medications [2]:

1. Review the diagnosis.
2. Check adherence to current medications: review prescription uptake, encourage families to bring the inhalers for appointments.
3. Check inhaler technique.
4. Check if spacer is being used.
5. Discuss and eliminate possible asthma triggers: cigarette smoke, pets, house dust mite and mould evidence.

### The Future for Paediatric Asthma

Lack of objective tests to diagnose asthma in young children continues to pose a significant challenge. However, frank discussion of these challenges and uncertainties with the families together with regular reviews, especially following a change in medication or recent worsening of symptoms, is crucial in supporting the families. Advice regarding minimising exposure to cigarette smoke, other pollutants and allergen avoidance measures should also be discussed. Although 95% of children with asthma can have good asthma control on small to medium doses of inhaled steroids, the recently published Nuffield report highlights suboptimal basic asthma care young people receive in the UK. The UK has the highest asthma related mortality in the western world.

Any child treated with 800 mcg beclomethasone equivalent, with more than one hospital admission or needing more than two courses of oral steroids in the past 12 months, should be referred to secondary care or the regional specialist paediatric asthma centre for further assessment and for consideration of novel therapies. Two biologicals are currently licensed in children > 6 years with severe asthma (Omalizumab, Mepolizumab).

The recently published *Lancet* commission [13] discusses redefining airway diseases based on pathology and objective tests, rather than purely on symptoms. More research is underway in understanding the pathology and identification of biomarkers for timely diagnosis and initiation of targeted therapies.

Whilst more research is undertaken in this field, regular education, medication reviews, asthma plans and timely referral, and communication between health care professionals remain the cornerstones for improving asthma care and for reducing morbidity and mortality.
